# Challenges and Advances in Nanotoxicology

**DOI:** 10.3390/nano4030766

**Published:** 2014-08-22

**Authors:** Robert L. Tanguay

**Affiliations:** Department of Environmental and Molecular Toxicology, Oregon State University, Corvallis, OR 97331-4003, USA, E-Mail: robert.tanguay@oregonstate.edu; Tel.: +1-541-737-6514; Fax: +1-541-737-6074

This Special Issue of *Nanomaterials* examines the potential for engineered nanomaterials to negatively impact biological systems and highlights some advances in evaluating key areas of their hazard potential. Nanomaterial science is evolving rapidly with the generation of more complex nanostructures with exciting potential applications. Keeping modern toxicology abreast of this innovation to the point that it guides a safer nanotechnology presents an equally exciting and eminently worthwhile challenge.

In this issue, Nogueira *et al.* [1] provide a critical review of current *in vitro* methods for measurement of nanostructure cytotoxicity. Cohignac *et al.* [2] review current understanding of the respiratory toxicity of nanomaterials and the possible role of autophagy, and Barna *et al.* [3] review the potential for carbon nanomaterial involvement in human granulomatous disease culled from epidemiological data of emergency first responders. This is followed by a new report from Totsuka *et al.* [4] that genotoxic effects of magnetite nanoparticles in the murine lung include oxidative stress- and inflammation related-DNA adduct formations, inflammatory cell infiltration and focal granulomatous formations. O'Shaughnessy *et al.* [5] report on the pulmonary sub-acute toxicity of double-walled carbon nanotubes in a murine model.

In medical nano-applications, Qiao *et al.* [6] report a protective role for low dose graphene oxide in normal cells against oxidative damage by X-ray radiation therapy. Papageorgiou *et al.* [7] address concerns about the safety of CoCr wear particles from synthetic spinal discs and suggest that CoCr nanoparticles could lead to significant secondary effects, such as neural tissue inflammation. On one of the newest nano-technology fronts, Saleh *et al.* [8] review how nanohybrids, conjugation of metal/metal oxides with carbonaceous nanomaterials and overcoating or doping of one metal with another, present both a new set of physicochemical properties and new challenges in hazard assessment. In ecotoxicology, Hanna *et al.* [9] offer new evidence that marine mussels have the potential to influence the fate and transport of CuO engineered nanoparticles and possibly act as a significant source of Cu to the marine benthos. In developmental toxicology, Harper *et al.* [10] report that carefully synthesized, high purity, peptide-functionalized AuNPs show high biocompatibility with the developing zebrafish embryo. Finally, in plant toxicology, Geisler-Lee *et al.* [11] report that AgNPs induce abiotic stress and cause reproductive toxicity in Arabidopsis, and Begum *et al.* [12] report substantial differential toxicity of multi-walled carbon nanotubes toward a variety of economically important crop species.
